# High-intensity interval training prevents muscle mass loss in overweight Chilean young adults during a hypocaloric-Mediterranean diet: a randomized trial

**DOI:** 10.3389/fnut.2023.1181436

**Published:** 2023-06-09

**Authors:** Matías Monsalves-Álvarez, Teresa Jiménez, Daniel Bunout, Gladys Barrera, Sandra Hirsch, Carlos Sepúlveda-Guzman, Claudio Silva, Juan M. Rodriguez, Rodrigo Troncoso, María Pía de la Maza

**Affiliations:** ^1^Instituto de Ciencias de la Salud, Universidad de O’Higgins, Rancagua, Chile; ^2^Laboratorio de Investigación en Nutrición y Actividad Física (LABINAF), Instituto de Nutrición y Tecnología de los Alimentos (INTA), Universidad de Chile, Santiago, Chile; ^3^Laboratorio de Ciencias del Ejercicio, Clínica MEDS, Santiago, Chile; ^4^Clínica Alemana, Santiago, Chile; ^5^Advanced Center for Chronic Diseases, Universidad de Chile, Santiago, Chile

**Keywords:** HIIT, body composition, Mediterranean diet, skeletal muscle, exercise

## Abstract

The hypocaloric Mediterranean diet (MD) mainly reduces fat mass but inevitably causes a loss of skeletal muscle mass. High-intensity interval training (HIIT) seems to have advantages in preserving muscle mass during a hypocaloric regime. Our study compares body composition and metabolic changes in overweight and obese Chilean women and men after 3 months of weight loss treatment with a Mediterranean-type hypocaloric diet, HIIT, or a combination of both. The study included 83 overweight or obese women and men between the ages of 25 and 50. The subjects were randomly assigned to one of the three intervention groups: (1) MD, (2) EX, and (3) MD + EX. Baseline and post-intervention measurements included: (a) body composition by dual-beam densitometry, muscle, and fat measurements by thigh ultrasound and computed tomography; (b) handgrip and quadriceps muscle strength; (c) exercise performance by peak oxygen consumption, peak load, work efficiency, and exercise energy expenditure; and (d) metabolic parameters. Out of 83 participants, the retention rate was 49% due to low compliance with the interventions. As expected, the MD group resulted in significantly greater weight loss (MD −7%, EX −0.6% and MD + EX −5.3%) and appendicular fat mass loss (MD −11.1%, EX −2.9, MD + EX −10.2%) but was associated with significant lean tissue loss (2.8%), which was prevented by HIIT (EX −0.1 and MD + EX −0.6%). Metabolic and glycoxidative parameters remained unchanged, irrespective of changes in body composition. Hypocaloric diets remain the most effective means to lose weight and body fat. However, it induces a loss of lean body mass when not accompanied by exercise training. This study shows that HIIT prevents the loss of muscle mass caused by a hypocaloric Mediterranean diet.

## Introduction

Healthy dietary patterns and exercise training have been proposed as primary components in the prevention and treatment of obesity and its comorbidities (1). Weight loss has been found to depend on different factors, such as the degree of energy deficit imposed by the diet, its duration, and macronutrient composition, which may directly influence adherence and treatment success (2–5). Among the diets, the Mediterranean Diet (MD), originally described by Ancel Keys, has been shown to promote a healthy metabolic profile mainly through its nutrient characteristics based on plants, unsaturated fats, fruits, and fiber while focusing on a low energy density, making it palatable and satiating (6). Interestingly, MD with limited amounts of carbohydrates has promoted differential mobilization of visceral fat depots and reduced cardiovascular risk factors in insulin-resistant patients. As with other diets, the weight loss seen with MD or low-calorie diets is mainly fat mass, but the weight loss inevitably affects skeletal muscle mass. It can range from ∼2 to 10%, depending on the age of the subjects (7, 8). This loss of lean muscle mass is likely to potentiate adverse effects on physical function and health (9).

Physical activity has a limited contribution to body fat reduction, even among different types of exercise (10–13). However, it is highly relevant for the preservation of muscle mass (7), weight maintenance (14), and improving cardiorespiratory fitness (15). Combining physical exercise with a hypocaloric diet induces slightly more weight loss in the short term (1.0–11.5 kg) (16). However, caloric restriction plus continuous aerobic training causes greater muscle mass loss than resistance exercise (17), suggesting that exercise modalities may play a role in maintaining muscle mass while dieting.

Although the benefits of even modest weight loss have been reported (18), it is unclear which intervention (diet vs. diet plus exercise or exercise alone) induces more favorable changes in body composition and chronic disease management in the long term. Weight maintenance is challenging due to adherence to behavioral changes and metabolic adaptations after weight loss. Therefore, researchers are looking for more effective and less time-consuming strategies that will hopefully increase commitment to healthy lifestyle changes.

High-intensity interval training (HIIT) has emerged as an alternative training modality that improves cardiorespiratory fitness and cardio-metabolic parameters in the healthy population (19) and in dieting obese patients (20, 21). However, few studies have addressed changes in segmental muscle mass and function and fat mass of different compartments after HIIT training (22–24), leading to potential differences when exercise training modalities are prescribed for the treatment of metabolic diseases.

The aim of the present study was to compare body composition and metabolic changes in overweight and obese men and women after 3 months of behavioral management for weight loss using a Mediterranean-type hypocaloric diet, HIIT, or a combination of both. We employed DEXA, abdominal CT scans, leg CT, and ultrasound (US) to analyze body compartments. Metabolic variables included serum glucose, lipoproteins, and insulin. We measured peak exercise oxygen consumption, workload, and handgrip and quadriceps muscle strength to evaluate functional changes. We hypothesized that adding HIIT to the dietary intervention would enhance its effects on body composition and metabolic profile. Our primary endpoints were the reduction of body fat and the maintenance of muscle mass. Secondary outcomes were the changes in the metabolic variables studied. This study was registered in Clinical Trials (NCT01793896); this manuscript refers to body composition and metabolic endpoints.

## Methods

### Subjects

The present study was a 3-month intervention trial. Inclusion criteria considered overweight (BMI 25 to 29.9 kg/m^2^) or obese (BMI greater than or equal to 30 kg/m^2^) men and women between the ages of 25 and 50 years who were interested in losing weight. At the same time, exclusion criteria included weight fluctuations (> 3 kg in the last 3 months), diabetes mellitus, neuromuscular or joint diseases, active smoking (>5 cigarettes/day), alcohol intake >30 g/day, and chronic diseases such as cancer, AIDS, or any organ failure. After signing a written informed consent, volunteers underwent an initial evaluation to rule out possible exclusion criteria and were then randomly assigned (according to a randomized number-based algorithm) to 1 of 3 interventions: a low-calorie Mediterranean-type diet (MD), supervised high-intensity interval training (HIIT) 3 times per week (EX), both interventions simultaneously (MD + EX). The study did not include a control group, but we performed a 3-month control period before randomization in 25 volunteers in which no intervention was prescribed.

### Study design

**Figure 1:**
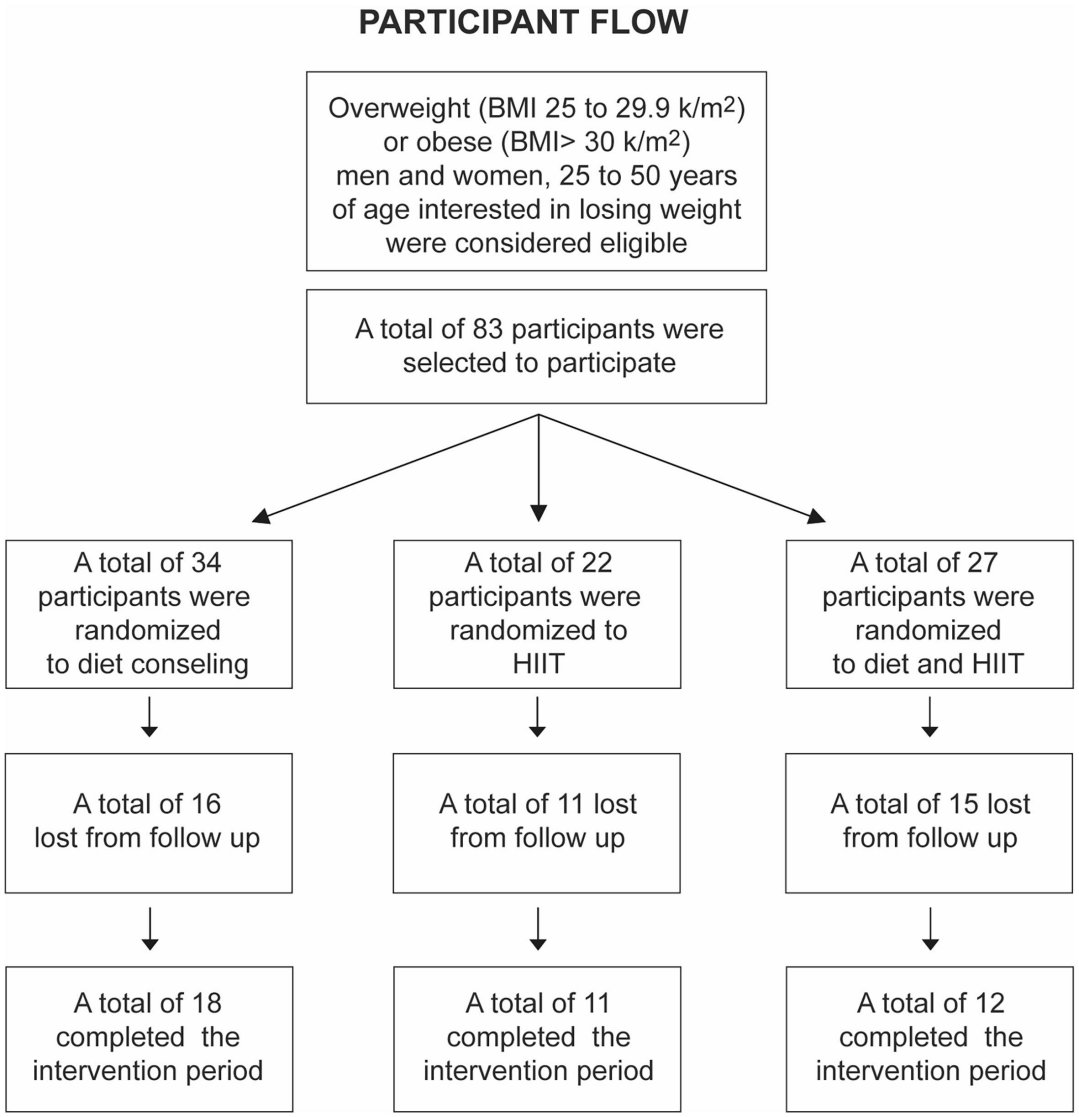
Participant flow diagram.

After the initial assessment, subjects were randomly assigned to receive one of the interventions for 3 months, repeating the same evaluation at the end of this period. A total of 83 participants were included and randomized into three different groups (Figure 1). **MD**: the subjects were prescribed a Mediterranean diet with a caloric intake of 20 Kcal/kg. The recommended foods in the MD, as previously described (25), were non-fat fermented milk, vegetables, legumes, fresh fruit, olive oil, and fish. Subjects were also instructed to avoid highly processed foods (such as breakfast cereals, cookies, cakes, powdered milk, and sausages). Wine consumption, which is typical of MD, was not recommended. Subjects were instructed to maintain physical activity or regular exercise without a supervised exercise plan and were checked weekly by the dietitian in charge. **EX:** High-Intensity Interval Training (HIIT) sessions were performed in three weekly sessions on a static bicycle (Spirit, model CU800, AR, United States), following the protocol of Gillen et al. (26). Subjects performed 10 repetitions of 1 min in each session, at 75% of the peak power achieved in the incremental test, with 1 min of rest cycling at 50 watts. This protocol was designed to achieve 85–90% of the maximum cardiac rate (HRmax) and included a 3-min warm-up and a 2-min recovery period, for a total of 25 min per session. **MED + EX**: subjects were enrolled in both interventions simultaneously.

### Dietary intake and Mediterranean score

Habitual dietary intake according to the survey of Uribarri et al. (27) and also the calculation of the Mediterranean Score using the “Aliméntate Sano Dietary Recall,” (28) available on the Internet (https://www.mifitbook.cl/que_es_fitbook.php).

### Blood samples

Fasting blood samples were collected by a venous puncture to obtain routine clinical biochemical tests (glucose, insulin, hemoglobin, lipoproteins, creatinine, prothrombin, and thyroid thyrotropin), analyzed in Vidaintegra laboratories.

### Body composition

Subjects were assessed for basic anthropometric measurements (height, weight, and waist circumference). Double-photon densitometry (DEXA) was evaluated for estimation of total body composition on Lunar Encore equipment (software 2011, version 13.60), with a minimal significant variability (MSV) at the 95% confidence level between two measurements of the same object or subject of 0.9% (29).

### Visceral and subcutaneous fat determination by computed tomography

Computed tomography (CT) at the L3 level to measure visceral and subcutaneous fat areas was performed in addition to the measurement of liver density as an indicator of fat infiltration, and CT at the same site of ultrasound measurements to estimate the rectus femoris muscle (RFM) area and density, in a Siemens Definition AS + tomograph with 128 detectors. The images were exported and segmented semi-automatically with the SliceOMatic software (version 5.0, Tomovision, Canada) by the region growth method and manual correction when necessary.

### Muscle mass cross-sectional area

Thigh ultrasound (US) was used to measure the cross-sectional area of the rectus femoris muscle (RFM), at the mid-level between the iliac crest and the patella using a General Electric Logiq ultrasonographer, with intra- and interrater errors of 3.2 and 7.4%, respectively (30).

### Physical performance

Quadriceps muscle strength was measured using a quadriceps table attached to a transducer, and handgrip strength was measured using a Therapeutic Instruments dynamometer (Clifton, NJ, USA), registering the best of three measurements on each limb (31). Cardiorespiratory fitness was determined by submaximal oxygen consumption (VO_2_) after an incremental test on a braked cycle ergometer using Sensormedics Vmax Encore 29 equipment. The incremental exercise test was started on a 15-watt ramp with 15-watt increments until volitional exhaustion at 60 rpm to measure VO_2peak_ (32). Gross work efficiency was calculated as the ratio of work rate to energy expenditure in joules multiplied by 100 at submaximal work rates (33).

### Energy expenditure

Actigraph (Actiheart®) was installed on all participants for 72 h for actigraphy and heart rate measurement. The devices were individually calibrated with the heart rate/energy expenditure curve obtained during the exercise calorimetry. Total energy expenditure (TEE), activity energy expenditure (AEE), and physical activity level (PAL) were determined (Actiheart software version 4.0.32, www.camtech.com).

### Statistical analysis

Baseline data distribution was analyzed using the Shapiro–Wilk test, with group comparisons performed using the Student’s *T*-test, one-way ANOVA, or Kruskal–Wallis, depending on their distribution. To analyze the effects of the interventions, we transformed the delta changes before and after the intervention into percentages and compared the means using a one-way ANOVA. We also assessed the changes in variables within and between treatment groups. To do this, we performed a multilevel mixed-effects linear regression for repeated measures on raw data. This method has been previously described to detect the time or treatment effect and the interaction between these two conditions (34). All analyses were performed using Stata 13.0 software. Table variables were expressed as mean ± SD, and median (range). Regarding power size calculation, according to our primary endpoint (loss of body fat) and based on a previous study (35), to obtain a 10% reduction in total fat after the intervention, eight participants per group were required to obtain results with an α of less than 0.05 and a power of 0.8 using a paired analysis.

## Results

As mentioned above, a total of 83 patients were initially selected according to the inclusion criteria, but 42 subjects failed to attend the exercise sessions or appointments with the RD, so we could not complete the final evaluation (body composition measurements and blood samples); therefore, the final sample for statistical analysis amounted to 41 (Figure 1). Table 1 shows all the variables studied by the group before the intervention. The groups were homogeneous in every variable at baseline, observing only the expected differences due to gender.

**Table 1:** Comparison of baseline variables between study groups.

Variable	MD (*n* = 18)	EX (*n* = 11)	MD+ EX (*n* = 12)	*p*
Sex (M/W)	9/9	4/7	4/8	
Age (years)	38 (25–50)	39 (26–49)	31.5 (23–48)	0.7088
Body composition and dietary recalls				
Weight (Kg)	79.9 (68.7–104.1)	79.9 (68.3–93.9)	77.9 (70.4–102.3)	0.9303
Body mass index (kg/mt^2^)	29.9 ± 1.7	29.5 ± 1.5	29.6 ± 1.6	0.7698
Waist circumference (cm)	98.6 ± 7.6	94.2 ± 6.9	96.2 ± 7.5	0.3088
Total lean body mass (kg)	42.0 (35.6–62.9)	40.6 (38.3–62.8)	45.6 (37.3–69.5)	0.9081
Appendicular lean body mass (kg)	18.8 (14.8–30.0)	18.7 (15.1–29.0)	21.0 (16.6–34.2)	0.8196
Total fat mass (kg)	32.6 ± 3.9	31.3 ± 4.6	31.1 ± 3.3	0.5269
Trunk fat mass (kg)	18.7 ± 2.8	17.7 ± 2.4	17.5 ± 2.6	0.4380
Appendicular fat mass (kg)	13.0 ± 2.4	12.3 ± 3.3	12.2 ± 2.1	0.6624
Mediterranean score *	5.3 ± 2.1	5.4 ± 1.7	4.7 ± 1.4	0.5937
Biochemistry				
Glucose (mg/dL)	90.0 ± 10.1	90.9 ± 7.6	88.3 ± 9.1	0.7977
Insulin (U/mL)	11.8 (4.8–36.1)	10.2 (6.6–28.8)	10.5 (4.5–34.2)	0.9453
HOMA-IR	2.7 (1.1–9.3)	2.2 (1.4–8.7)	2.2 (1.2–3.9)	0.3011
Total cholesterol (mg/dL)	189 (126–362)	194 (111–223)	202 (149–223)	0.5691
HDL cholesterol (mg/dL)	47 (26–77)	43 (35–63)	52 (32–91)	0.6348
LDL cholesterol (mg/dL)	107.7 ± 29.4	105.9 ± 24.2	101.0 ± 43.2	0.8601
Tryglicerydes (mg/dL)	148 (45–1,372)	134 (34–353)	139 (62–385)	0.8722
Muscle strength, ultrasound, and CT scans				
Right-hand strength (kg)	29 (17–52.5)	25 (16–49.5)	32.8 (18–48)	0.5208
Left-hand strength (kg)	25.5 (15–49.5)	25 (15–50.5)	29.5 (15–47.5)	0.4176
Right quadriceps strength (N)	350.9 ± 86.8	370.0 ± 80.3	415.7 ± 128.8	0.2248
Left quadriceps strength (N)	373.6 ± 79.3	377.3 ± 95.5	424.1 ± 123.7	0.3540
US–right RFM area (cm^2^)	21.2 ± 3.4	23.7 ± 2.6	24.2 ± 5.7	0.1066
US–left RFM area (cm^2^)	21.2 ± 3.5	22.6 ± 2.8	24.0 ± 5.4	0.1540
CT–left RFM area (cm^2^)	25.9 ± 3.4	27.1 ± 3.4	26.8 ± 5.0	0.7053
CT–left RFM.Density (HU)	50.0 ± 3.2	52.4 ± 3.0	50.8 ± 2.2	0.1414
CT–hepatic density (HU)	54.4 (9.5–66.6)	61.3 (30.1–71.5)	54.1 (17.5–62.2)	0.0624
CT–total Abdominal Fat area (cm^2^)	492.5 ± 79.0	454.7 ± 58.2	433.0 ± 65.2	0.0849
CT–abdominal visceral fat area (cm^2^)	118.8 (51.1–310.4)	134.4 (59.6–234.6)	106.5 (73.8–237.1)	0.9270
CT–abdominal subcutaneous fat area (cm^2^)	348.3 ± 66.9	309.6 ± 86.3	296.5 ± 43.9	0.1137
*Exercise tests*				
VO_2_peak (ml/Kg/min)	17.3 (10.8–32.1)	17.2 (11.3–34.3)	20.3 (14.6–31.2)	0.2839
Maximal load (W)	143.2 ± 56.5	155.3 ± 45.4	169.0 ± 55.7	0.4467
Exercise time (Min)	7.7 (4.4–14.6)	7.9 (4.5–19.7)	7.0 (5.2–13.9)	0.7361
Gross work efficiency (W/J)	7.7 ± 1.5	8.0 ± 1.4	7.8 ± 1.0	0.9093
Activity energy expenditure (KCal)	706 (176–2,931)	521 (194–2,171)	776 (196–1773)	0.7726
Physical activity level (METS)	1.5 (1.2–2.8)	1.4 (1.3–2.4)	1.6 (1.3–2.1)	0.8941

DEXA, dual-energy x-ray absorptiometry/RFM, rectus femoris muscle/CT, computed tomography/US, ultrasound / W, watts/J, joules. Values are mean ± SD or median (range) according to the distribution of the variables. Analysis performed by Kruskall–Wallis 2 or one-way ANOVA, depending on the distribution of variables. Significant when *p* ≤ 0.05. *Mediterranean score according to the “Aliméntate Sano” dietary survey, where scores 0–4 = low, 4–9 = moderate, and 9–14 = high (14 points being optimal). **Dietary AGEs according to a semiquantitative dietary recall, where < 10 kU/d = low AGE intake, >10 < 16 kU/d = moderate AGE intake and > 16 kU/d = elevated AGE intake.

### Brief and intense exercise attenuates appendicular lean body mass loss induced by low-calorie MD

Exercise has been shown to prevent the loss of muscle mass induced by caloric restriction (9). In Table 2, we show that after 3 months of intervention, there was a significant reduction (expressed as %) in body weight, BMI, and waist circumference in MD and MD + EX when compared to EX alone (*p* < 0.0001). Regarding body composition, appendicular LMB, trunk, and total FM were also significantly different in the MD group compared to the other two groups (*p* = 0.007, 0.03, and 0.03, respectively). Regarding biochemical parameters, no statistical differences were observed, despite a decrease in glucose and insulin in the EX and MD + EX groups (−1.5 and − 3.1% reduction, respectively). Interestingly, visceral and subcutaneous fat determined by CT showed that MD alone resulted in a significant reduction in abdominal visceral and subcutaneous fat area (*p* = 0.02 and 0.04), although MD + EX had a − 15.4% and − 15.8% reduction on the same CT measured areas, respectively. We found no difference in exercise testing between groups, but VO_2peak_ and workload showed percentage improvements in EX and MED + EX groups.

**Table 2:** Changes in study variables after 3-months of intervention.

	MD (*n* = 18)	EX (*n* = 11)	MD + EX (*n* = 12)	*p*
Body composition and dietary recalls				
Weight (%)	−7.2 ± 3.6**α**	−0.6 ± 3.2	−5.3 ± 2.7**α**	**< 0.0001**
Body mass index (%)	−7.5 ± 3.6**α**	−0.4 ± 3.4	−6.1 ± 2.9**α**	**< 0.0001**
Waist circumference (%)	−7.8 ± 4.5**α**	−1.9 ± 3.3	−7.4 ± 3.6**α**	**0.0009**
DEXA-total lean body mass (%)	−2.8 ± 2.8**α**	−0.1 ± 2.3	−0.6 ± 2.1	0.0153
DEXA-appendicular lean body mass (%)	−3.4 ± 4.4**α**	5.8 ± 12.5	−1.6 ± 2.7	**0.0070**
DEXA–total fat mass (%)	−11.7 ± 13.3	−3.8 ± 4.7	−13.5 ± 8.0	0.0770
DEXA–trunk fat mass (%)	−14.6 ± 13.8	−4.0 ± 4.9	−15.2 ± 8.9	**0.0317**
DEXA–appendicular fat mass (%)	−11.1 ± 10.3**α**	−2.9 ± 5.3	−10.2 ± 7.3	**0.0375**
Mediterranean score	71.5 ± 84.3	32.4 ± 64.6	69.6 ± 60.4	0.3397
*Biochemistry*				
Glucose (%)	1.4 ± 8.1	−1.5 ± 7.0	−3.1 ± 4.8	0.2224
Insulin (%)	16.7 ± 76.8	−2.8 ± 34.9	−12.8 ± 33.9	0.3691
Homa–IR (%)	21.7 ± 108.5	30.6 ± 140	5.3 ± 64.5	0.8474
Total cholesterol (%)	−0.6 ± 18.8	−1.1 ± 19.3	−6.1 ± 8.5	0.6472
HDL cholesterol (%)	2.5 ± 16.4	3.4 ± 15.6	0.4 ± 10.6	0.8830
LDL cholesterol (%)	15.8 ± 54	2.2 ± 53.0	12.6 ± 43.2	0.7816
Tryglicerydes (%)	2.2 ± 67.1	−4.3 ± 36.2	−1.6 ± 34.4	0.9456
Muscle strength, ultrasound, and CT scans				
Right-hand strength (%)	1.6 ± 19.8	1.9 ± 6.6	6.3 ± 11.5	0.6789
Left-hand strength (%)	6.9 ± 17.1	7.0 ± 22.9	3.2 ± 22.8	0.8689
Right quadriceps strength (%)	5.0 ± 16.2	7.4 ± 21.2	3.8 ± 27.3	0.9224
Left quadriceps strength (%)	−0.8 ± 11.4	6.1 ± 17.1	1.0 ± 16.5	0.4871
US–right RFM area (%)	5.2 ± 14.0	10.6 ± 12.0	6.9 ± 9.4	0.5239
US–left RFM area (%)	3.1 ± 13.7	7.9 ± 9.6	7.9 ± 10.5	0.4503
CT–left RFM area (%)	−0.1 ± 12.5	4.6 ± 9.9	5.5 ± 14.4	0.4338
CT–left RFM.DENSITY (%)	2.2 ± 5.2	−1.1 ± 6.2	1.2 ± 7.6	0.4428
CT–Hepatic density (%)	25.0 ± 45.9	1.8 ± 15.6	17.4 ± 44.4	0.3508
CT–total abdominal fat area (%)	−18.1 ± 13.7**α**	−4.1 ± 6.7	−15.4 ± 13.6	**0.0215**
CT–abdominal visceral fat area (%)	−15.5 ± 25.0	1.7 ± 15.7	−12.5 ± 28.2	0.1969
CT–abdominal subcutaneous fat area (%)	−18.0 ± 13.0**α**	−6.9 ± 6.2	−15.8 ± 10.6	**0.0432**
Exercise tests				
VO2 peak (%)	8.2 ± 28.4	20.0 ± 32.3	24.6 ± 29.9	0.3110
Maximal load (%)	9.9 ± 37.0	14.9 ± 24.4	22.3 ± 26.8	0.5939
Exercise time (%)	8.6 ± 43.8	13.6 ± 41.4	9.3 ± 26.5	0.9435
Gross work efficiency (%)	1.5 ± 21.9	−5.6 ± 30.4	6.7 ± 27.8	0.5328
Activity energy expenditure (%)	11.9 ± 35.6	−4.3 ± 52.5	20.9 ± 45.4	0.5466
Physical activity level (%)	3.5 ± 7.6	0.9 ± 22.2	4.1 ± 14.8	0.8744

DEXA, dual-energy X-ray absorptiometry / RFM, rectus femoris muscle/CT, computed tomography/US, ultrasound/W, watts/J, joules. Values represent % change from baseline (means ± SD), analyzed by one-way ANOVA. Significant when *p* < 0.05–for post-hoc analysis, **α =** significantly different from EX Group.

### Muscle performance is improved during hypocaloric-MD diets with high-intensity intervals

Preserving skeletal muscle performance is essential to maintaining function at any age (36). Table 3 shows the variables that exhibited significant changes after the intervention according to the mixed-effects linear regression for repeated measures method. Here, we found a lack of weight and total fat loss in the EX group (79.7 to 77 kg and 29.4 to 29.3 kg, respectively), while maintaining lean body mass in MD + EX (∼45.6 kg), and an increase in VO_2peak_ and maximal load (W) associated with the inclusion of a HIIT modality in MED + EX (7.4 ml·kg·min and 54 W, respectively). Results did not change significantly when both exercise groups were combined with the calorie-restricted group. In conclusion, caloric restriction-induced greater fat and muscle loss. However, the incorporation of short-interval HIIT prevented muscle mass loss while increasing muscle strength and aerobic capacity as determined by VO_2peak_.

**Table 3:** Intergroup differences before and after the interventions.

	MD (*n* = 18)		EX (*n* = 11)		MD + EX (*n* = 12)		*Post-hoc*
	Initial	Final	Initial	Final	Initial	Final	
Weight (Kg)	79.9 (74.6–85.3)	72.6 (67.5–77.8)	79.9 (71.8–87.7)	77 (73.7–86.2)	77.9 (72–93.6)	73.7 (67.8–86.4)	**α**
DEXA–total lean body mass (Kg)	42 (38.1–52.3)	40.6 (37.2–50.7)	40.6 (39.7–57.2)	42.8 (39.1–56.9)	45.6 (38.6–57.2)	45.6 (38.8–56)	**#**
DEXA–total fat mass (Kg)	32.7 (30.6–35.7)	29.5 (26.9–31.4)	29.4 (28.2–33.2)	29.3 (26.7–30.8)	31.6 (29.4–33)	27.2 (25.5–28.6)	**α**
DEXA–trunk fat mass (Kg)	18.1 (16.2–20.2)	16.5 (14.5–17.4)	18.2 (14.9–18.9)	16.5 (15.3–17.6)	17.4 (15.9–18.9)	14.9 (13.2–15.8)	**α**
DEXA-appendicular lean body mass (Kg)	18.8 (16.7–25)	17.9 (16.7–23.6)	18.7 (18–22.8)	20.1 (18–26.5)	21 (17.2–26.4)	20.5 (17.3–25.9)	**α**
DEXA-appendicular fat mass (Kg)	12.9 (11.3–14.7)	12.3 (9.4–13.5)	12.5 (8.8–14.6)	10.9 (9–13.7)	12.7 (10.1–13.9)	11.2 (9.3–12.3)	**α**
CT-total Abdominal fat area (cm^2^)	456.6 (442.6–550.5)	421.3 (339.4–452.8)	440.7 (408.1–509.5)	402.1 (381.2–459.8)	415.9 (381.9–483.3)	373 (324.1–419.4)	**α**
CT-abdominal subcutaneous fat area (cm^2^)	333.5 (308.3–414.6)	293 (239.8–351.7)	298.2 (239.5–382.7)	269.3 (214–329)	303.3 (256.6–327.3)	256.9 (199.9–292.8)	**α**
VO2 peak (mL/Kg/min)	17.3 (13–21.5)	16.9 (15.1–20.2)	17.2 (13.8–25.6)	24.8 (18.8–27)	20.3 (17.2–25.5)	27.7 (20.3–31.7)	**&**
Maximal load (W)	135 (100–181)	130 (110–184)	156 (118–195)	156 (132–183)	160 (120.5–207)	217 (166–243.5)	**&**

DEXA, dual-energy X-ray absorptiometry / RFM, rectus femoris muscle / CT, computed tomography/ US, ultrasound / W, watts / J, joules. Values represent the median (p25-p75) at baseline and after the interventions, analyzed by mixed-effects linear regression for repeated measures method. Significant when *p* < 0.05–For *post-hoc* analysis, **α =** EX group significantly different, # = MD group significantly different, and & = MD + EX group significantly different.

## Discussion

In this study, we used a hypocaloric MD, HIIT 3 times per week, and diet plus training to induce changes in body composition and metabolic parameters. We analyzed the results by protocol, including the 41 compliant men and women. Baseline variables did not differ between compliant and 42 excluded subjects (data not shown). As in previous studies, we observed that three short weekly sessions of HIIT attenuated calorie restriction-induced fat and lean body mass loss. Exercise is often included in lifestyle intervention programs to promote metabolic flexibility and prevent and treat metabolic diseases (37). Muscle loss associated with even a small and short-term caloric restriction must be emphasized because intermittent dieting is frequent in sedentary obese patients, increasing the risk of future sarcopenia and weight regain (38). These findings are relevant because we have previously detected lower muscle mass and strength in our population, even in young adults (39).

One of the main issues regarding the effectiveness of exercise and dietary treatments in patients with chronic conditions is adherence, which in our experience is around 50% (35, 40, 41). We assessed compliance with exercise by registering attendance at scheduled training sessions. As patients usually justify their lack of time, HIIT seemed to be a more time-efficient and less time-consuming strategy. However, in this new study, we observed similar retention rates, which puts pressure on the development of new strategies to achieve adherence in studies that involve efforts such as dietary changes and exercise.

Recently, the randomized PERIMED-plus trial showed that in men and women with metabolic syndrome who followed a Mediterranean diet for 12 months, 40% of the participants reported reductions in weight, waist circumference, blood pressure, and cholesterol with a low dropout rate (42), showing the effectiveness of this dietary pattern in the long term. When analyzing body composition changes by DEXA, it is evident that the hypocaloric diet (MD) induced a significantly greater loss of fat mass and a decrease in lean body mass (especially appendicular). These results were not confirmed by US or CT measurements of femoral muscle mass, suggesting that these measurements were less sensitive because they were localized to only one muscle area compared to the four extremities in DEXA. As expected, the training method employed was well tolerated. However, it did not induce changes in handgrip or quadriceps strength, as can be observed with strength training modalities mediated by mechanical loading and increased muscle protein synthesis pathways (43, 44). Exercise alone did not induce any changes in body composition or muscle parameters, unlike those reported by Blue et al. who demonstrated that 3 weeks of HIIT increased leg vastus lateralis cross-sectional area (CSA) by 14% in obese and overweight participants, but without any caloric restriction (45). However, exercise is essential for preserving muscle mass during hypocaloric diets.

Our results confirm that HIIT can preserve lean mass during a negative energy balance. Interestingly, it has been shown that the mitochondrial and myofibrillar protein synthesis responses are increased after high intensity (above 60% watt_max_) when compared to low-intensity bouts of aerobic training (30% watt_max_) in young men (46). This may explain why our subjects (trained at 75% watt_max_) maintained their lean mass despite a diet-induced energy deficit. It is worth mentioning that the protein intake in our protocol was set at 1 g/kg body weight, which seems to be sufficient, at least for these young obese patients, to preserve muscle in this type of exercise regime with a hypocaloric MD (47).

We observed a barely significant but clinically relevant change in VO_2peak_ that could be explained by factors such as an inadequate training dose (frequency, intensity, volume), insufficient to promote changes in fitness, and interindividual variability. Some investigations have demonstrated significant interindividual variability without improvements in cardiorespiratory fitness in response to typical exercise doses, attributed partly to genetic variants and exercise prescriptions (48, 49). A growing body of evidence indicates that changes following a regular training program vary among individuals, particularly depending on initial fitness levels, which in the present study were low. Hence, not all subjects will respond in the same way to a given dose of physical training. These subjects are known as non-responders. It is likely that our prescribed amount of HIIT (a fixed workload of 75% watt_max_) was insufficient to induce changes in cardiovascular fitness in most of our volunteers. It may have been more convenient to prescribe training based on respiratory thresholds, as suggested by several authors (40, 41). Thus, non-responders to training should be considered more trained according to inadequate physiological parameters (40, 41, 50) and not as refractory to exercise programs.

Surprisingly, despite a more than 5% change in body weight, a nearly 12% decrease in fat mass, and a significant reduction in abdominal fat after hypocaloric dieting, no differences were observed in metabolic and oxidative stress indicators, contradicting clinical and scientific results. It is possible that adherence to the Mediterranean dietary pattern allowed a loss in total body fat and liver fat. However, no correlation was observed between an increase in the Mediterranean Score and changes in metabolic parameters such as glucose, insulin, HOMA-IR, and lipoprotein levels. Subjects who started the intervention with two or more features of the metabolic syndrome (waist circumference, elevated triglycerides, low HDL cholesterol, or elevated HOMA-IR) behaved similarly. We do not explain these results except for the high variability of laboratory parameters (especially HOMA-IR), which could have been amplified by the small sample size. A recent meta-analysis showed that only aerobic exercise reduced insulin levels and HOMA-IR (51). Another multicenter study using HIIT showed significant changes in these parameters in prediabetic men and women (52), although employing a lower-volume, higher-intensity protocol (5-by-1 min HIT, at ~125% VO_2max_ cycling intensity). In a small sample of healthy subjects, long-term endurance training reduced triglyceride, glucose, and creatinine levels and increased superoxide dismutase activity (53). Lipoprotein profiles have improved through high-intensity aerobic and moderate resistance training (54, 55). Some studies have demonstrated that HIIT induces favorable metabolic changes in overweight and obese adults, while others have shown no apparent changes (56). These differences may be attributed to patient selection and specific training modalities, among other variables.

This study has limitations: a small sample size, due to low adherence to the three interventions, despite every effort to increase it, and a short intervention period. The reduced sample also precluded the analysis of men separately from women, who may have responded differently to diet and training. Our results may not apply to other age groups or groups with a wider gender distribution. With respect to dietary prescriptions, we could not ascertain these subjects’ actual protein intake because dietary recall proved limited. However, this study also has significant strengths, such as the various methods used to ensure proper body composition assessment and the measurement of several metabolic and functional parameters to detect changes induced by the interventions. Also, the exclusion of non-compliant volunteers allows for the determination of whether positive changes or a lack of effects occurred and were not a consequence of statistical error.

In conclusion, this study confirms that the best way to reduce body fat is to reduce energy intake. However, this can induce rapid muscle mass loss, which can be avoided by short sessions of HIIT. According to our results, fat mass loss seems to have a less relevant role in metabolic parameters than expected.

## Data availability statement

The raw data supporting the conclusions of this article will be made available by the authors, without undue reservation.

## Ethics statement

The studies involving human participants were reviewed and approved by Ethics Committee of the Institute of Nutrition and Food Technology (INTA), Universidad de Chile. The patients/participants provided their written informed consent to participate in this study.

## Author contributions

MM, MM-Á, TJ, and DB conceived and designed the study. MM-Á, TJ, JR, GB, CS-G, CS, and DB performed the experiments. MM-Á, DB, MM, SH, and RT analyzed the data. MM-Á, DB, RT, and MM interpreted the data. MM-Á, TJ, DB, RT, and MM drafted the manuscript, which was reviewed by all authors. All authors contributed to the article and approved the submitted version.

## Funding

This work was supported by the Chilean Agencia Nacional de Investigación y Desarrollo (ANID): MM #1130284 and MM-A #11230186.

## Conflict of interest

The authors declare that the research was conducted without any commercial or financial relationships construed as a potential conflict of interest.

## Publisher’s note

All claims expressed in this article are solely those of the authors and do not necessarily represent those of their affiliated organizations, or those of the publisher, the editors and the reviewers. Any product that may be evaluated in this article, or claim that may be made by its manufacturer, is not guaranteed or endorsed by the publisher.
